# Notes and Notices

**Published:** 1883-06

**Authors:** 


### NOTES AND NOTICES.

Removal: J. W. Thomson, M. D., from Springfield, Mass., to 645 Chapel Street, New Haven, Conn.

Dr. William Gallupe, an old and true homœopathist, died recently at his home in Bangor, Maine.

The Homœopathic Medical Society of Wisconsin will meet at Madison, June 12th–14th, in joint session with the Western Academy of Homœopathy. An interesting and instructive meeting is expected.

Neuralgia from Tea Drinking.—Dr. Francis states in a paper read before the Harveian Society, London, that the habit of tea drinking at and between meals, now so common in England, renders people peculiarly liable to neuralgia.

Biblical View of Allopathic Treatment.—The first instance where physicians are mentioned in the Bible is II Chronicles xvi, 12. It is not flattering to allopathy: "And Asa, in the thirty-ninth year of his reign, was diseased in his feet until the disease was exceedingly great, yet in his disease he sought not the Lord, but the physicians. And Asa slept with his fathers."

Pessary in the Rectum.—Dr. H. L. Turney relates the history of a lady patient who had been treated by a homœopath for retroverted uterus. Dr. Turney was summoned to her one night in haste and found the patient in great pain. He examined her and found a large size Hodge pessary skillfully lodged in the rectum.—*Nashville Journal of Medicine and Surgery.*

Hypertrophy of the Humerus.—Miss Dora Huls, of Reading, Pa., aged seventeen years, bled to death through the bursting of an artery in her right arm above the elbow, where the bone had grown until it became over two and a half feet in circumference and weighed forty-two pounds. Last spring she visited Jefferson College, when Dr. Gross told her the only remedy was the amputation of her arm, but she said she would rather die than submit to the operation. The bone was enlarging two years. She was unable to walk the last three weeks.

Horseflesh as Food for the Sick.—In the *Bulletin de Therapeutique* Dr. Beaumetz describes the uses of "powdered horse," a new meat preparation employed in what is known as the artificial alimentation of the sick. It is obtained by drying chopped horseflesh at a temperature of 120°, and then reducing it to an impalpable powder. It is gray in color, and has an odor like pate de foie gras. The doctor pronounces it very nourishing, and its fineness makes assimilation easy. Under its use the laziest stomachs are said to resume their functions, and appetite returns.

A Sixth Sense.—It is announced that the distinguished physicist, Sir William Thompson, ascribes to a man six senses, namely, those of *force*, heat, sound, light, taste, and smell.

We apprehend that this announcement comes rather late in the day, as American text-books have for some time past taught this doctrine. As an instance we may mention *The Human Body*, by Dr. H. Newell Martin, of the Johns Hopkins University, published in 1881. In this work Dr. Martin states, "We commonly distinguish five senses, those of sight, sound, touch, taste, and smell," and he then suggests that "temperature" should be added. —*Medical Record.*

Goats and Homœopathy.—It is a pleasure to learn that the goat is an animal which responds well to homœopathic remedies. In Surrey, England, there is a goat farm where *Guilielmuz capricornis* is raised and milked for the alleged benefit of the babies of London. A visitor says: "The goats, young and old, appear clean and perfectly healthy; their bright, hairy coats are subjected to curry-combing; no troublesome foot disease demands attention as in the case of sheep, and any internal ailments are promptly and successfully dealt with by homœopathic medicines, of which the manager, Mr. Farrer, speaks with the greatest confidence and satisfaction." We should like very much to have Mr. F. report to us some cases. No doubt infinitesimal doses will affect a credulous man, but not a sensible goat.—*Journal of Comparative Medicine.*

